# Supplementary material for: The distinct metabolic phenotype of lung squamous cell carcinoma defines selective vulnerability to glycolytic inhibition
Source: Nat Commun. 2017 May 26;8:15503. doi: 10.1038/ncomms15503 (PMC5458561; doi:10.1038/ncomms15503)
Supplement: Supplementary Information — Supplementary Figures and Supplementary Tables [file ncomms15503-s1.pdf]

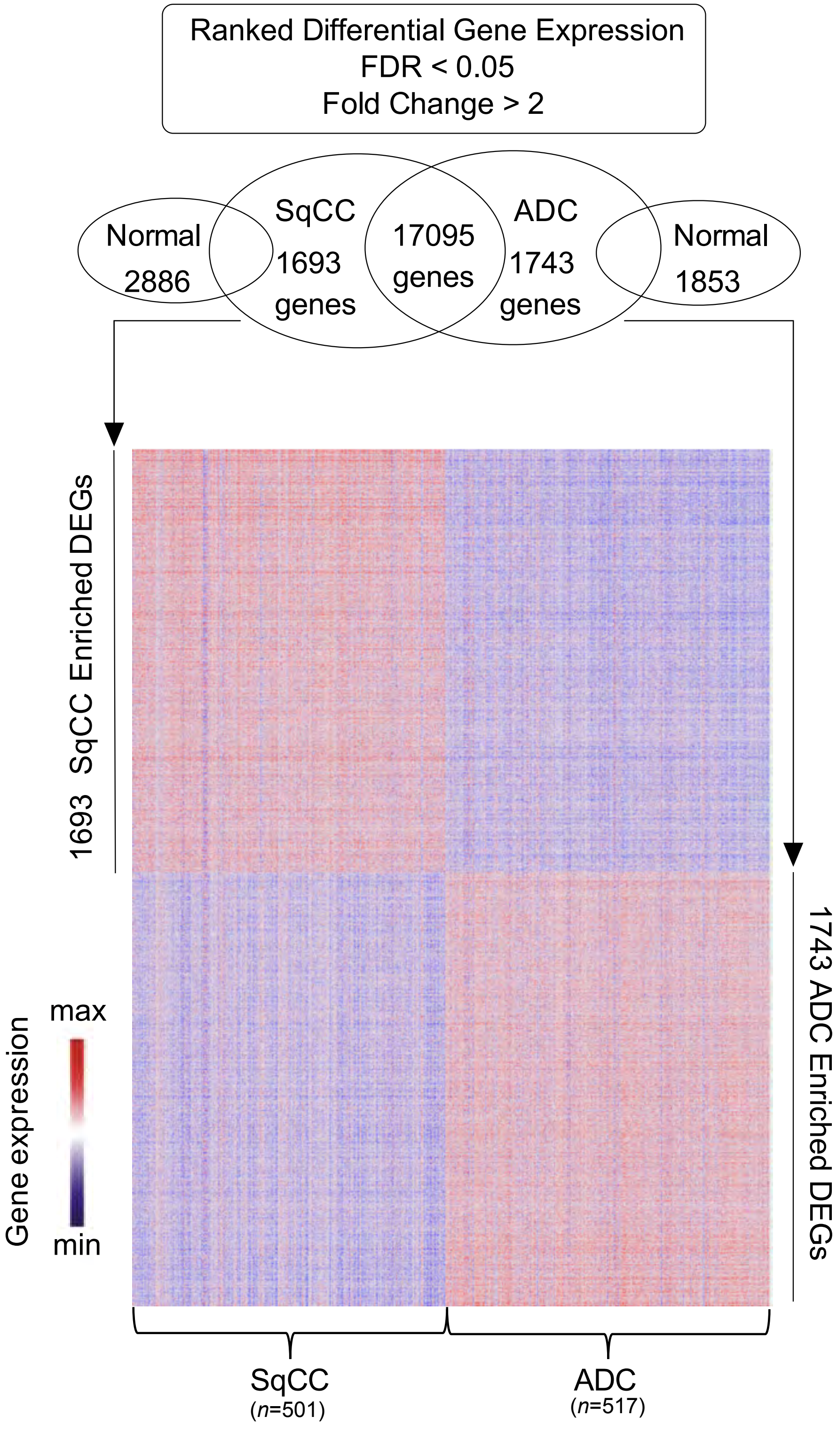

Supplementary Figure 1 Schematic representation of differential gene expression analysis. Level three gene expression data from the TCGA lung SqCC and ADC cohorts were ranked by differential expression (in normalized TPM), P<0.05 with false discovery rate (FDR) < 5%, and a minimum fold change of two between SqCC and ADC. Identified DEGs were then compared to normal tissue to ensure normal/tumor differential expression.

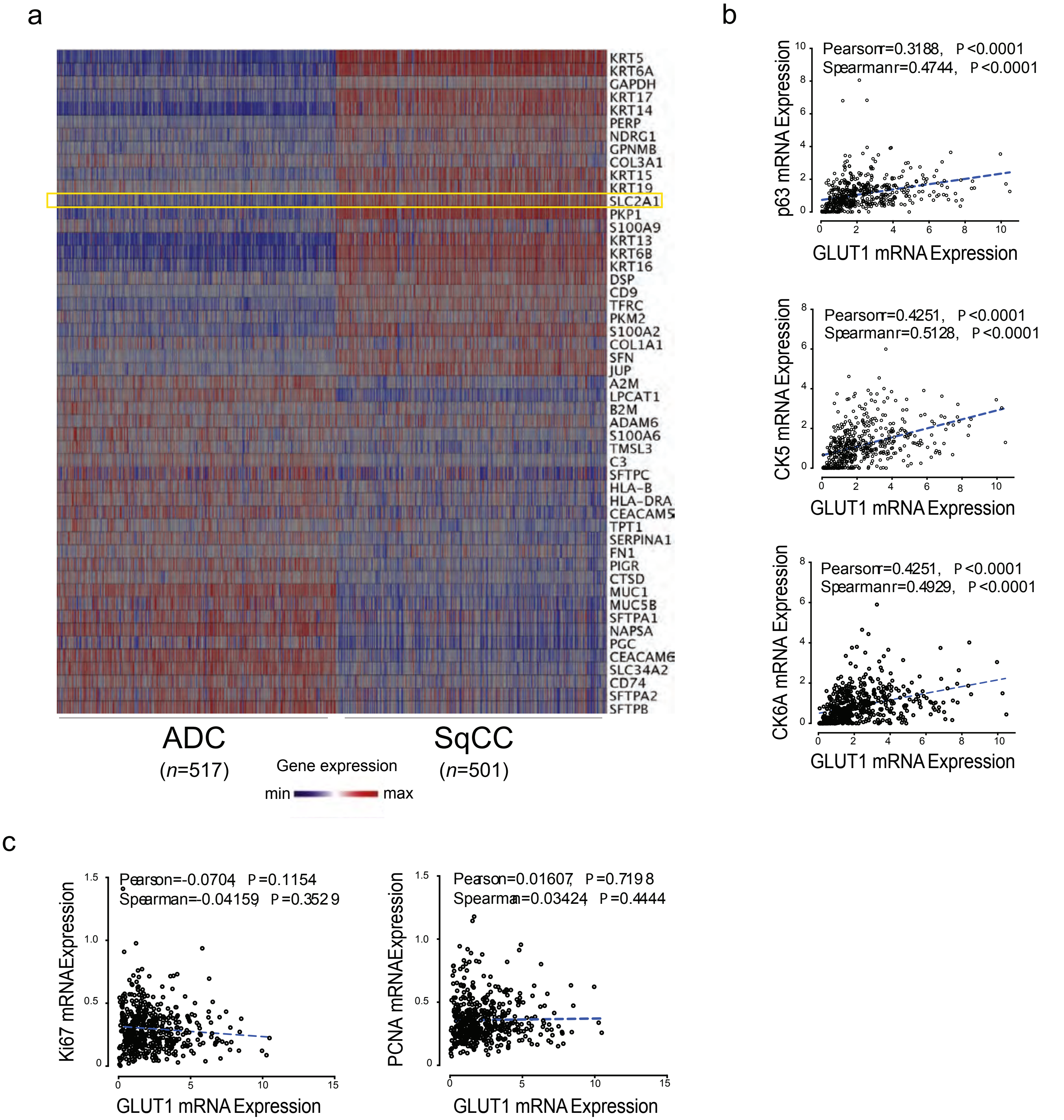

Supplementary Figure 2 GLUT1 identified as highly associated with lung SqCC. (**a**) Heatmap depicting the expression distribution of the most significantly differentially expressed genes between 501 SqCC and 517 ADC tumor samples from the TCGA mRNA sequencing data. (**b**, **c**) Correlative analysis of GLUT1 mRNA expression with p63, Cytokeratin 5, and Cytokeratin 6A (**b**), and Ki67, and PCNA (c) mRNA expression in TCGA SqCC samples. Pearson and Spearman R-values and probabilities are presented for correlations. Gene expression values are normalized TPM.

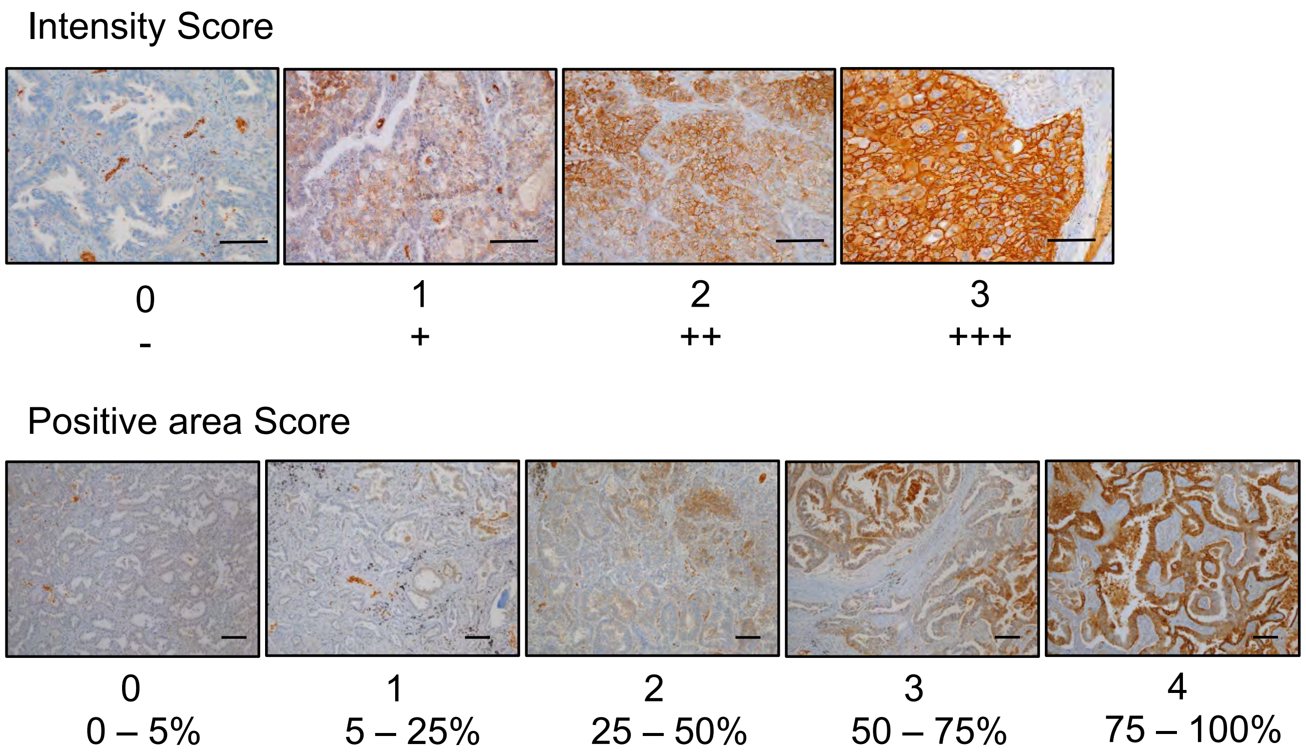

Supplementary Figure 3 Histopathological scoring criteria used for analysis of GLUT1 staining in human clinical tumor tissue. SqCC and ADC samples were scored by pathologists based on GLUT1 intensity and % positive area. Scale bar, 50 µm.

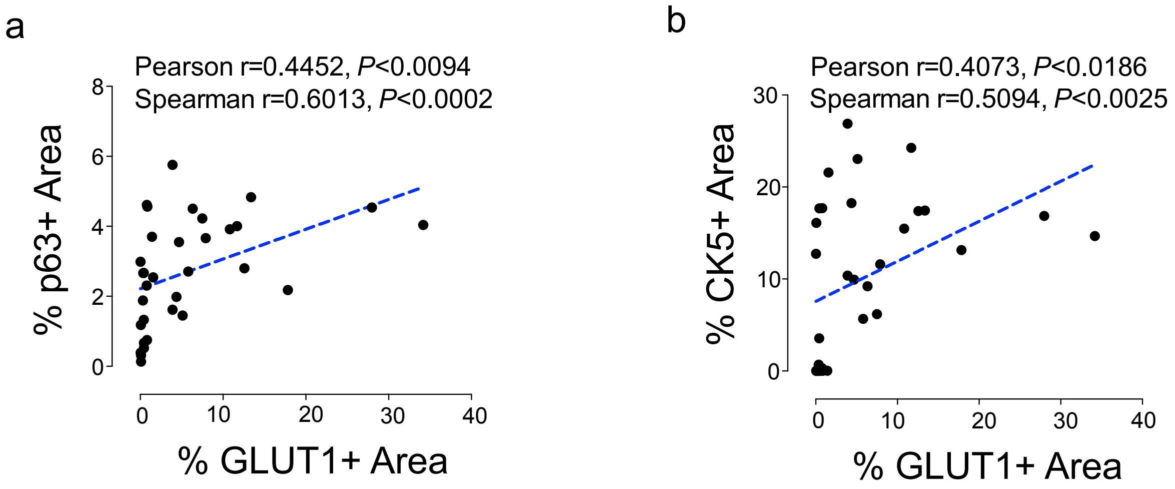

Supplementary Figure 4 High GLUT1 expression is associated with SqCC phenotype in NSCLC tissue microarray samples. (**a**, **b**) Correlative analysis between SqCC-specific molecular markers p63 (**a**) and CK5 (**b**) with GLUT1 expression in human SqCC tissue microarray tumor samples (n=34). Pearson and Spearman R-values and probabilities are presented for correlations.

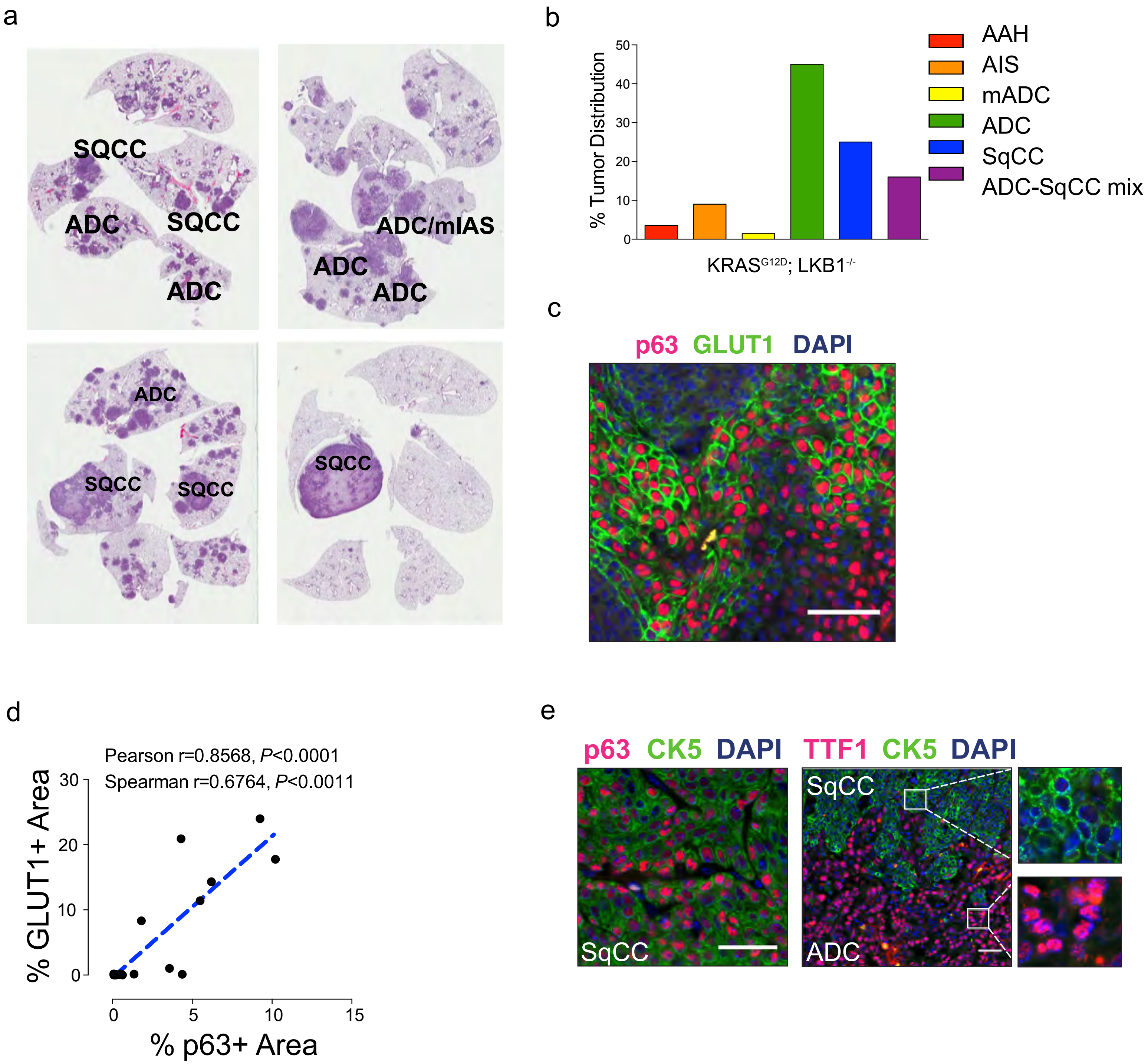

Supplementary Figure 5 Characterization of GLUT1 expression in the spectrum tumors produced by the KL murine model of NSCLC. (**a**) Representative H&E images of tumor-bearing KL mouse lungs. (**b**) % distribution of histological phenotypes observed in the KL murine model of NSCLC. AAH, atypical adenomatous hyperplasia; AIS, Adenocarcinoma in situ; mADC, mucin adenocarcinoma; ADC, adenocarcinoma; SqCC, squamous cell carcinoma. (**c**) Representative immunofluorescent co-staining of p63 and GLUT1 in KL SqCC tumors. Scale bar, 50 μm. (**d**) Correlation of GLUT1 expression with p63 expression in KL murine model of NSCLC (n=11). Pearson and Spearman R-values and probabilities are presented for correlations. (**e**) Representative IF images of SqCC markers p63 and CK5 (left), and ADC marker TTF1 in neighboring KL SqCC and ADC tumors (right). Scale bar, 50 μm.

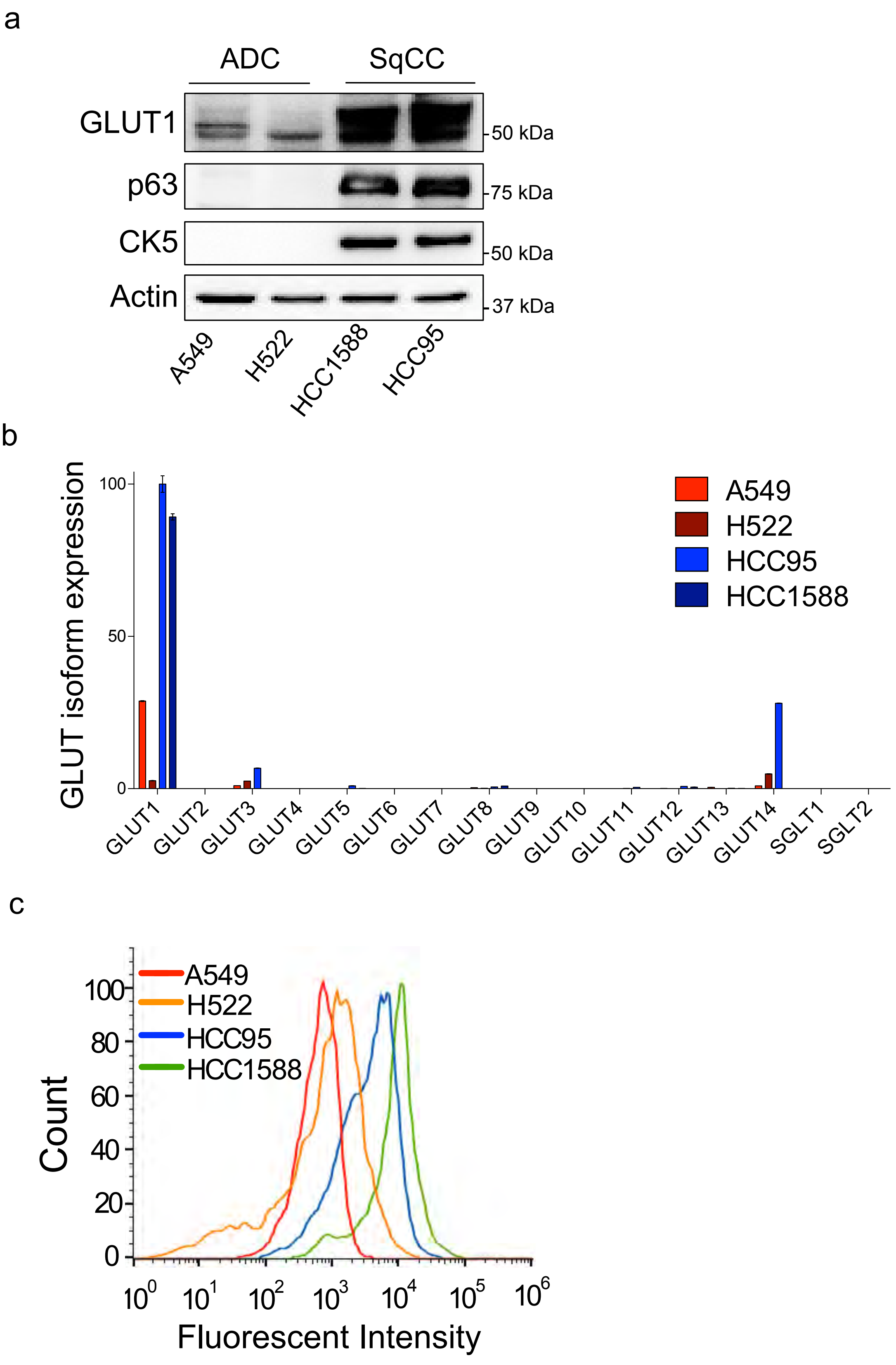

Supplementary Figure 6 GLUT1 is highly elevated in lung SqCC cell lines and contributes to increased glucose uptake. (**a**) Immunoblot analysis of SqCC markers, p63 and CK5, with GLUT1 in A549 and H522 (ADC), and HCC1588 and HCC95 (SqCC). (**b**) qPCR analysis of the mRNA expression of glucose transporters, GLUT1-14 and two sodium glucose transporters SGLT1 & 2, in NSCLC cell lines. GLUT1 mRNA expression was normalized relative to -actin mRNA expression. (n=4 from at least two biologically independent experiments). Error bars represent the mean s.e.m. (**c**) Representative flow cytometry histogram of fluorescent glucose uptake in ADC and SqCC cell lines.

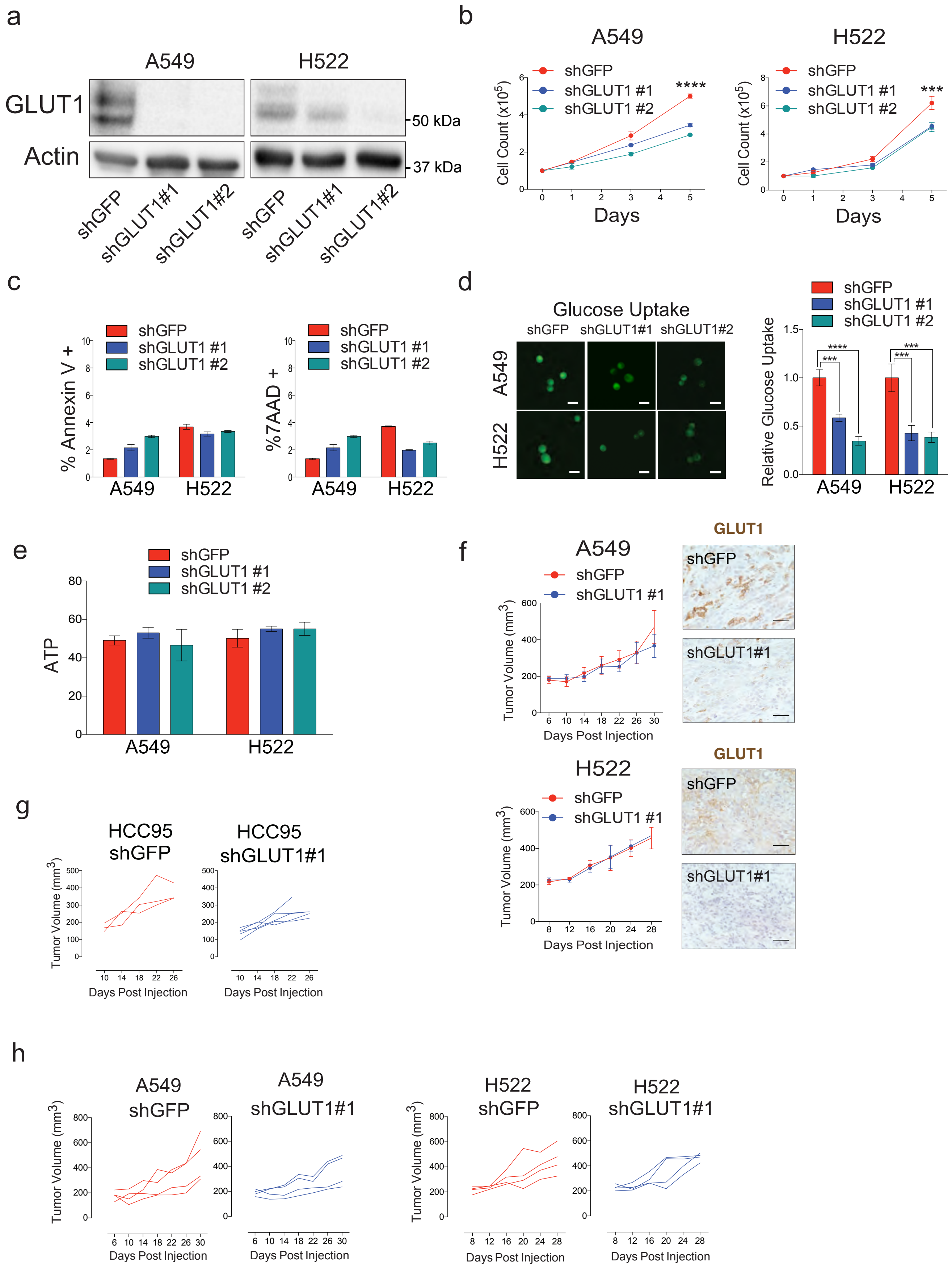
a
A549
H522
GLUT1
Actin
50 kDa
37 kDa
shGFP
shGLUT1#1
shGLUT1#2
b
A549
H522
shGFP
shGLUT1 #1
shGLUT1 #2
Cell Count (x10^5)
Days
****
***
c
% Annexin V +
%7AAD +
d
Glucose Uptake
Relative Glucose Uptake
e
ATP
f
Tumor Volume (mm^3)
Days Post Injection
GLUT1
g
HCC95 shGFP
HCC95 shGLUT1#1
h
A549 shGFP
A549 shGLUT1#1
H522 shGFP
H522 shGLUT1#1

Supplementary Figure 7 GLUT1 knockdown does not affect lung ADC cell viability and in vivo tumor growth. (**a**) Immunoblot analysis of GLUT1 expression in control shGFP and shGLUT1 A549 and H522 ADC cells. (**b**) In vitro proliferation of control shGFP and shGLUT1 A549 and H522 ADC cells (n=6 each group from two biologically independent experiments). Two-way ANOVA, ****P<0.0001. (**c**) Cell viability was assayed via Annexin-V and 7-AAD staining in control shGFP and shGLUT1 A549 and H522 ADC cells (n=2 each group from two biologically independent experiments). ANOVA. (**d**) Representative fluorescent images (left), and quantification (right) of fluorescent glucose uptake in shGFP and shGLUT1 A549 and H522 cells (n=3, 6-9 images were captured in each group for quantification). Two-tailed t-test, ****P<0.0001, ***P<0.001. Scale bar, 25 μm . (**e**) Comparison of relative intracellular ATP levels between shGFP and shGLUT1 A549 and H522 cells (n=6 each group from two - three biologically independent experiments). Two-tailed t-test. (**f**) In vivo tumor growth (left) and representative GLUT1 immunoblot (right) of shGFP and shGLUT1 A549 and H522 xenograft tumors (n=4 for each group). Scale bars, 50 μm. (**g**, **h**) Individual growth curves of shGFP (left) and shGLUT1 (right) xenograft tumors of HCC95 SqCC cells (**g**) and A549 and H522 ADC cells (**h**) All error bars represent the mean s.e.m.

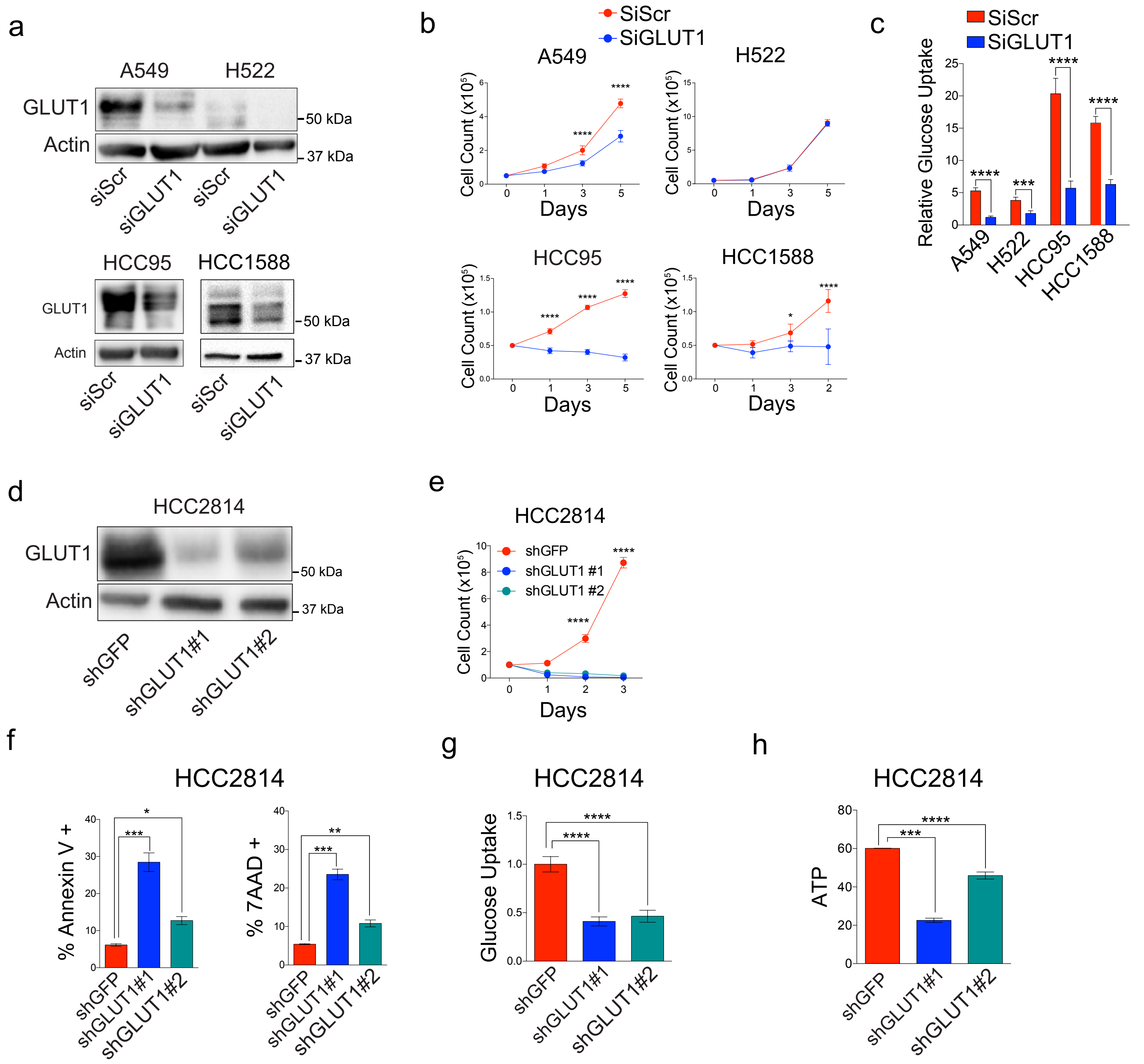

Supplementary Figure 8 Transient GLUT1 knockdown inhibits in vitro proliferation of lung SqCC while SqCC cell line HCC2814 is susceptible to GLUT1 knockdown. (**a**) Immunoblot analysis of GLUT1 expression in control siScramble (siScr) and siGLUT1 ADC (A549 and H522) and SqCC (HCC95 and HCC1588) cells. (**b**) In vitro proliferation of control siScr and siGLUT1 ADC (A549 and H522) and SqCC (HCC95 and HCC1588) cells (n=4 each group from two biologically independent experiments). Two-way ANOVA, ****P<0.0001, *P<0.05. (**c**) Quantification of fluorescent glucose uptake in siScr and siGLUT1 ADC (A549 and H522) and SqCC (HCC95 and HCC1588) cells (n=2, 8 images were captured in each group for quantification). Two-tailed t-test, ****P<0.0001, ***P<0.001. (**d**) Immunoblot analysis of GLUT1 expression in control shGFP and shGLUT1 HCC2814 cells. (**e**) In vitro proliferation of control shGFP and shGLUT1 HCC2814 cells (n=2 each group from two biologically independent experiments). Two-way ANOVA, ****P<0.0001. (**f**) Cell viability was assayed via Annexin-V and 7-AAD staining in control shGFP and shGLUT1 HCC2814 cells (n=2 each group from two biologically independent experiments). ANOVA, ****P<0.0001, ***P<0.001, *P<0.05 (**g**) Fluorescent glucose uptake in shGFP and shGLUT1 HCC2814 cells (n=2, 8 images were captured in each group for quantification). Two-tailed t-test, ****P<0.0001. (**h**) Comparison of relative intracellular ATP levels between shGFP and shGLUT1 HCC2814 cells (n=3 each group from two biologically independent experiments). Two-tailed t-test, ****P<0.0001, ***P<0.001.

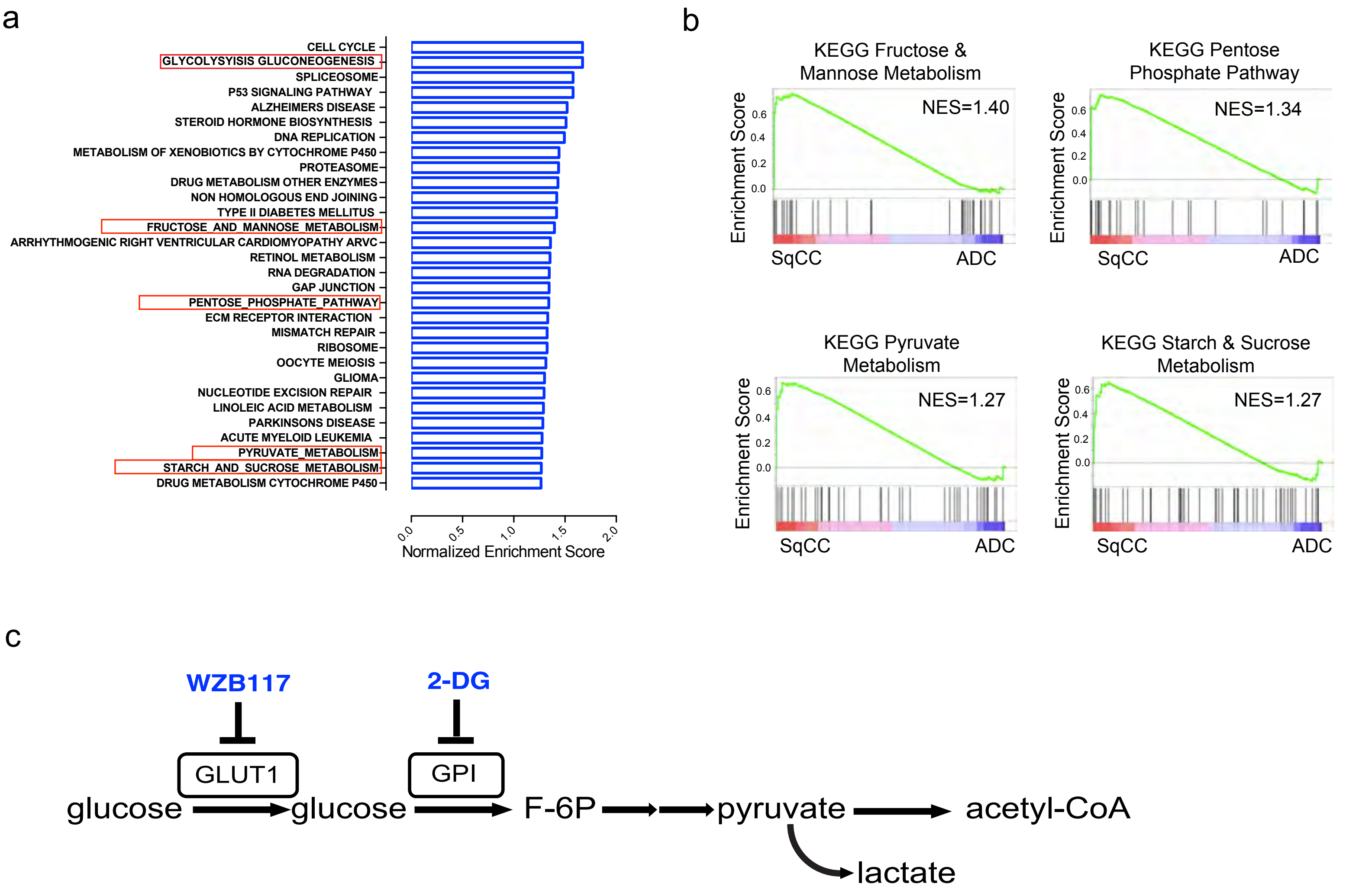

Supplementary Figure 9 Elevated glycolytic and branching pathways in TCGA lung SqCC patient tumor samples. (**a**) The top 30 KEGG gene sets enriched in SqCC patients (n=501) compared to ADC patients (n=517) by GSEA. Red box highlights glycolysis or gene sets which branch from glycolysis. (**b**) GSEA mountain plots for an enrichment of genes belonging to the KEGG gene sets for fructose and mannose metabolism, pentose phosphate pathway, pyruvate metabolism, and starch and sucrose metabolism in SqCC gene expression profiles compared to ADC. NES, normalized enrichment score. (**c**) Schematic diagram of chosen glycolytic targets and inhibitors in the current study.

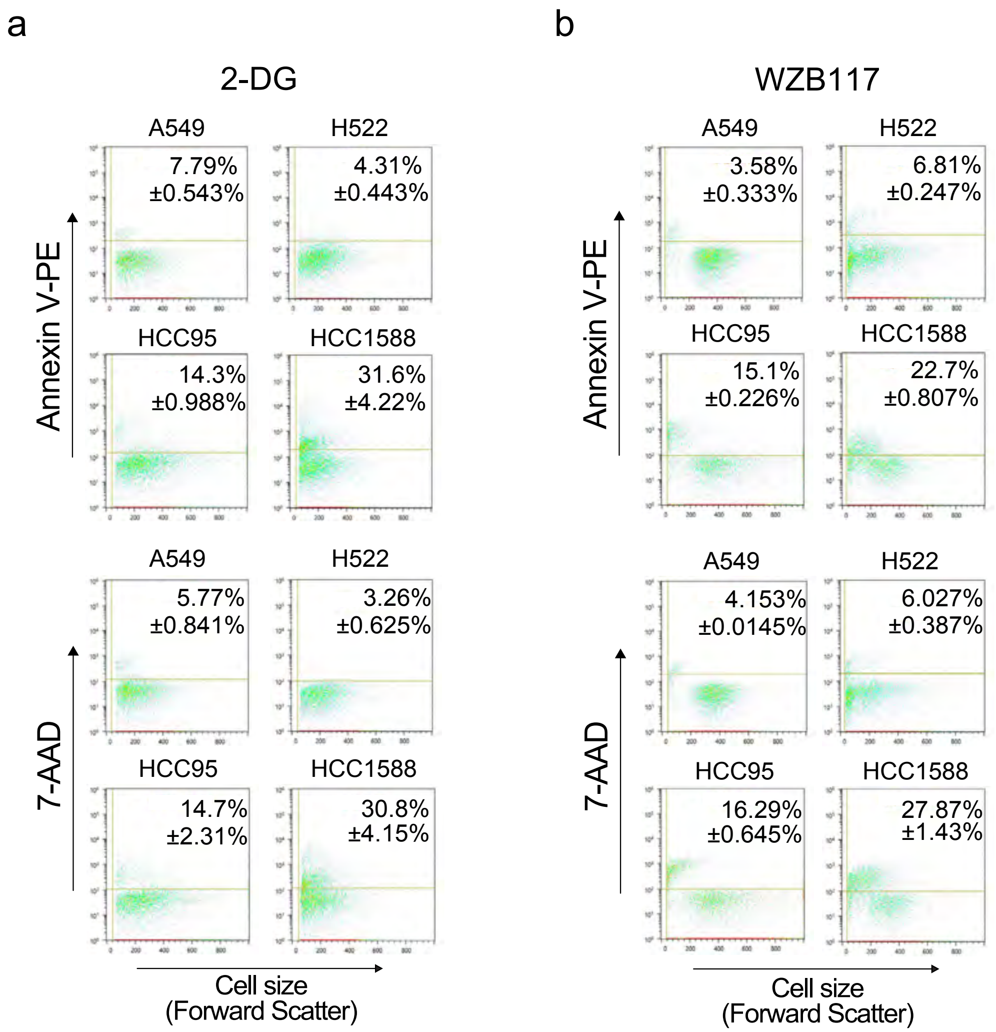

Supplementary Figure 10 Lung SqCC cells are selectively susceptible to glycolytic inhibition. (**a**, **b**) Flow cytometry of cell viability assay using Annexin V-PE and 7-AAD after treatment with glycolytic inhibitor, 2-DG (25mM) (**a**) and GLUT1 inhibitor, WZB117 (50μM) (**b**) for 48-72 hours (n=5 each group from at least three biologically independent experiments).

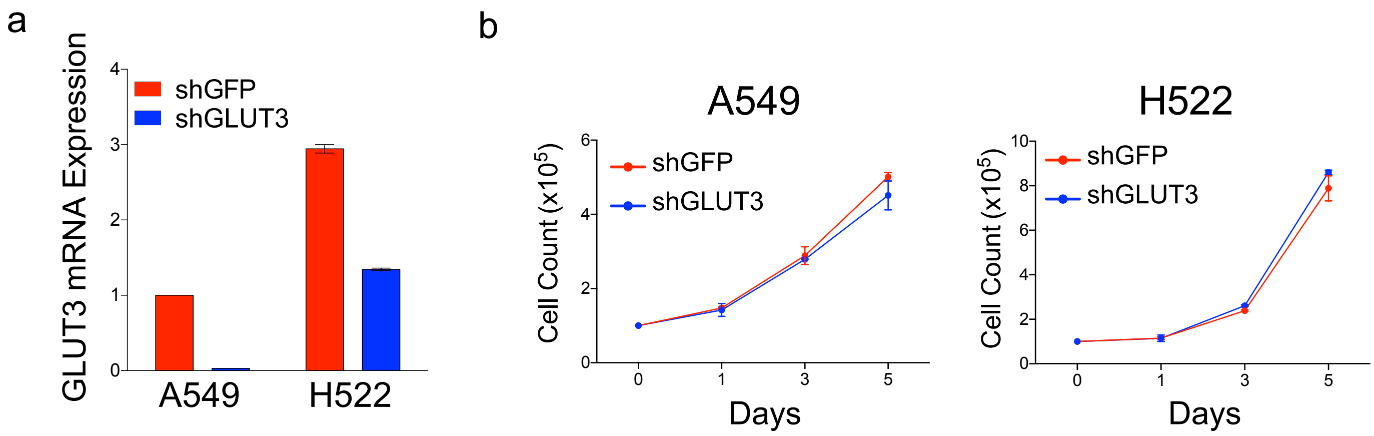

Supplementary Figure 11 GLUT3 does not functionally contribute to lung ADC in vitro proliferation. (**a**) qRT-PCR analysis of GLUT3 expression in shGFP control and shGLUT3 A549 and H522 (n=2 from 2 biologically independent experiments). (**b**) In vitro proliferation of control shGFP and shGLUT3 A549 and H522 ADC cells (n=4 each group from two biologically independent experiments). Two-way ANOVA.

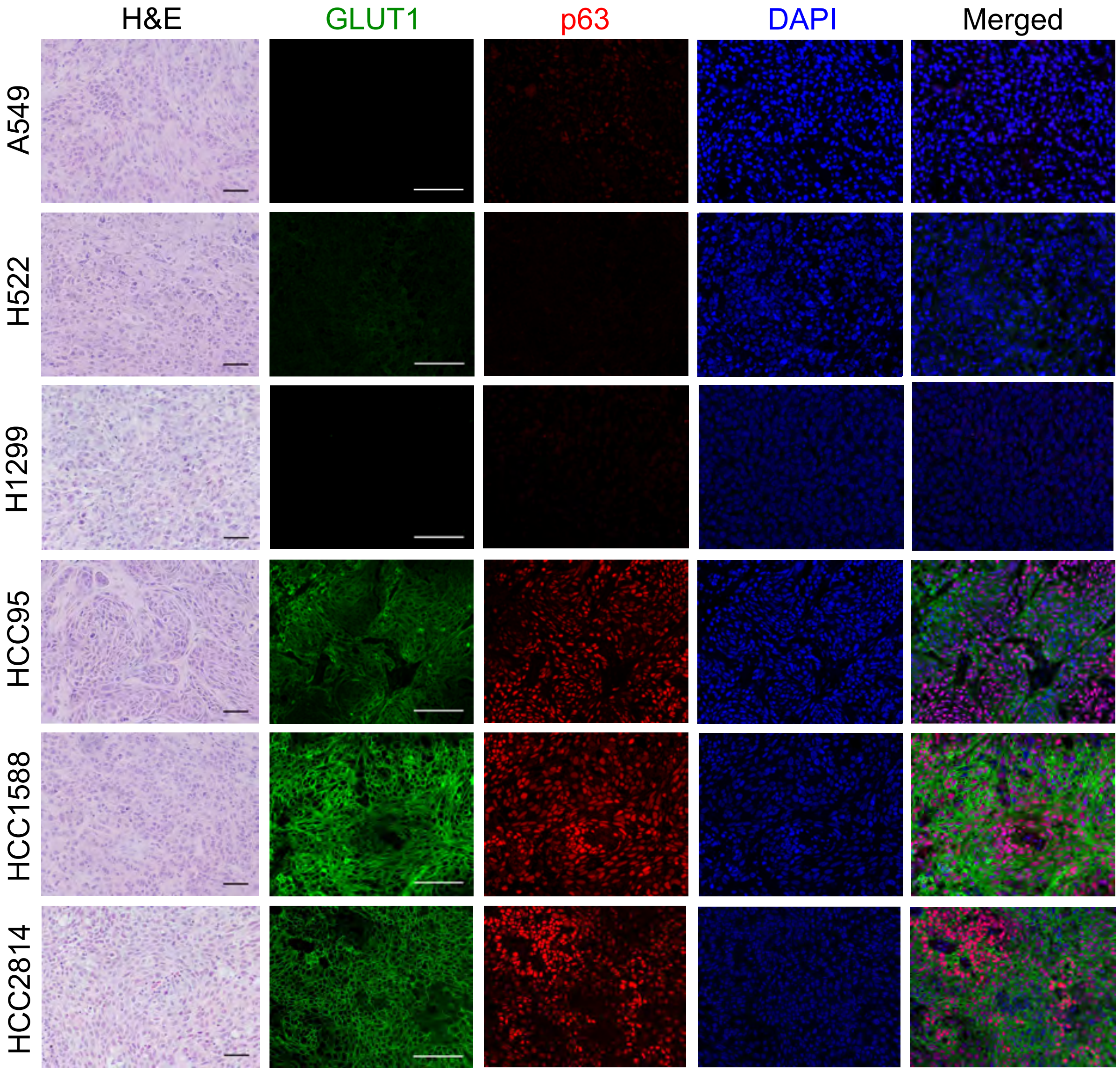

Supplementary Figure 12 NSCLC xenograft tumors. Representative H&E and immunofluorescent images of GLUT1 and p63 in serial sections from ADC (A549 and H522) and SqCC (HCC95 and HCC1588) xenograft tumors. Scale bar, 100 μm.

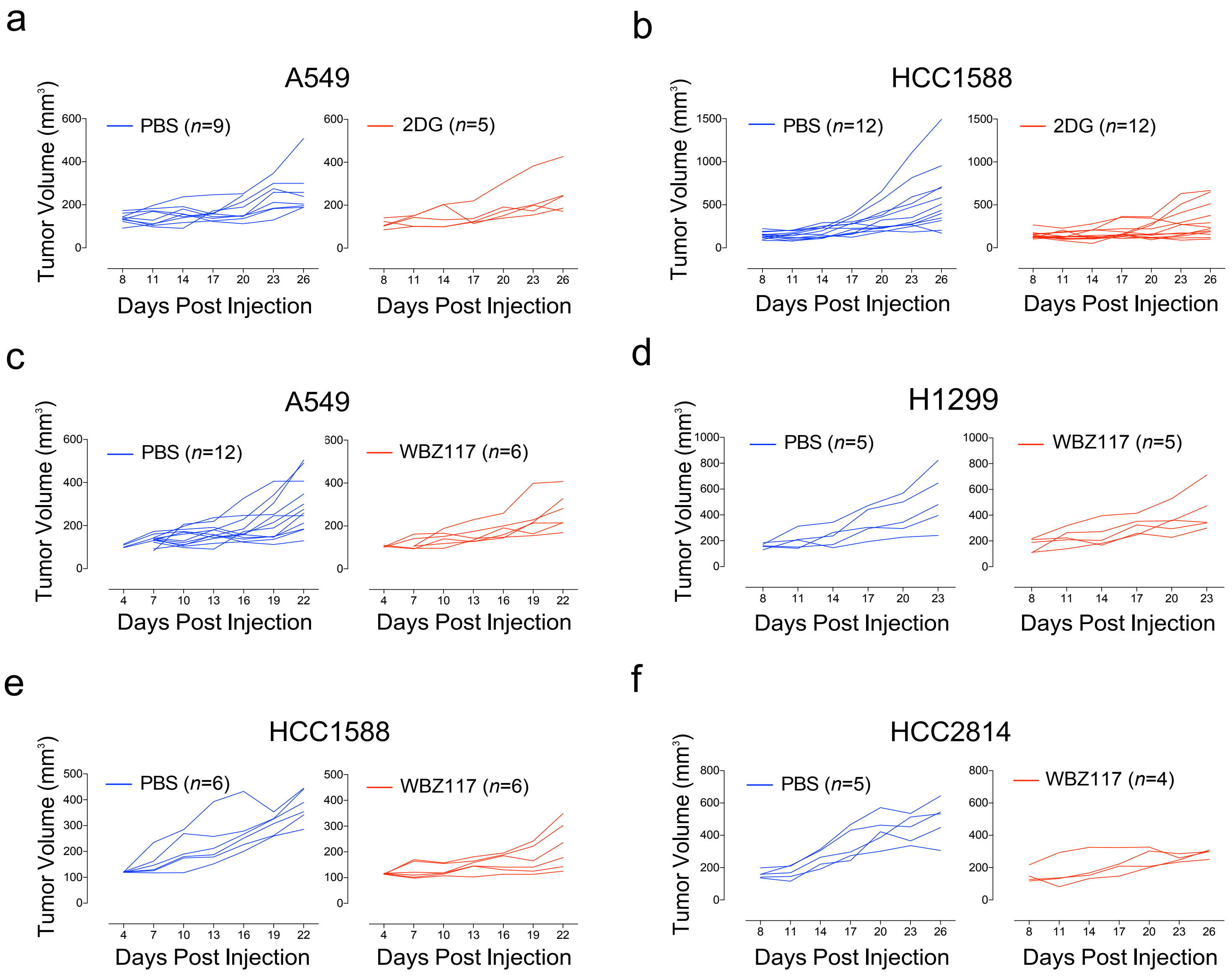

Supplementary Figure 13 Growth of individual xenograft tumors under vehicle, 2-DG, or WZB117 treatment. (**a**, **b**) Individual xenograft tumor growth of ADC A549 (PBS, n=9; 2-DG, n=5) (**a**) and SqCC HCC1588 (PBS, n=12; 2-DG, n=12) (**b**) tumors treated with PBS as vehicle or glycolytic inhibitor 2-DG (500mg/kg, once daily). (**c**, **d**) Individual xenograft tumor growth of ADC A549 (PBS, n=11; WZB117, n=6) (**c**) and H1299 (PBS, n=5; WZB118, n=5) (**d**) tumors treated with PBS/DMSO as vehicle or GLUT1 inhibitor WZB117 (10mg/kg, once daily) (**e**, **f**) Individual xenograft tumor growth of SqCC HCC1588 (PBS, n=7; WZB117, n=5) (**e**) and HCC2814 (PBS, n=5; WZB117, n=4) (**f**) tumors treated with PBS/DMSO as vehicle or GLUT1 inhibitor, WBZ117 (10mg/kg, once daily).

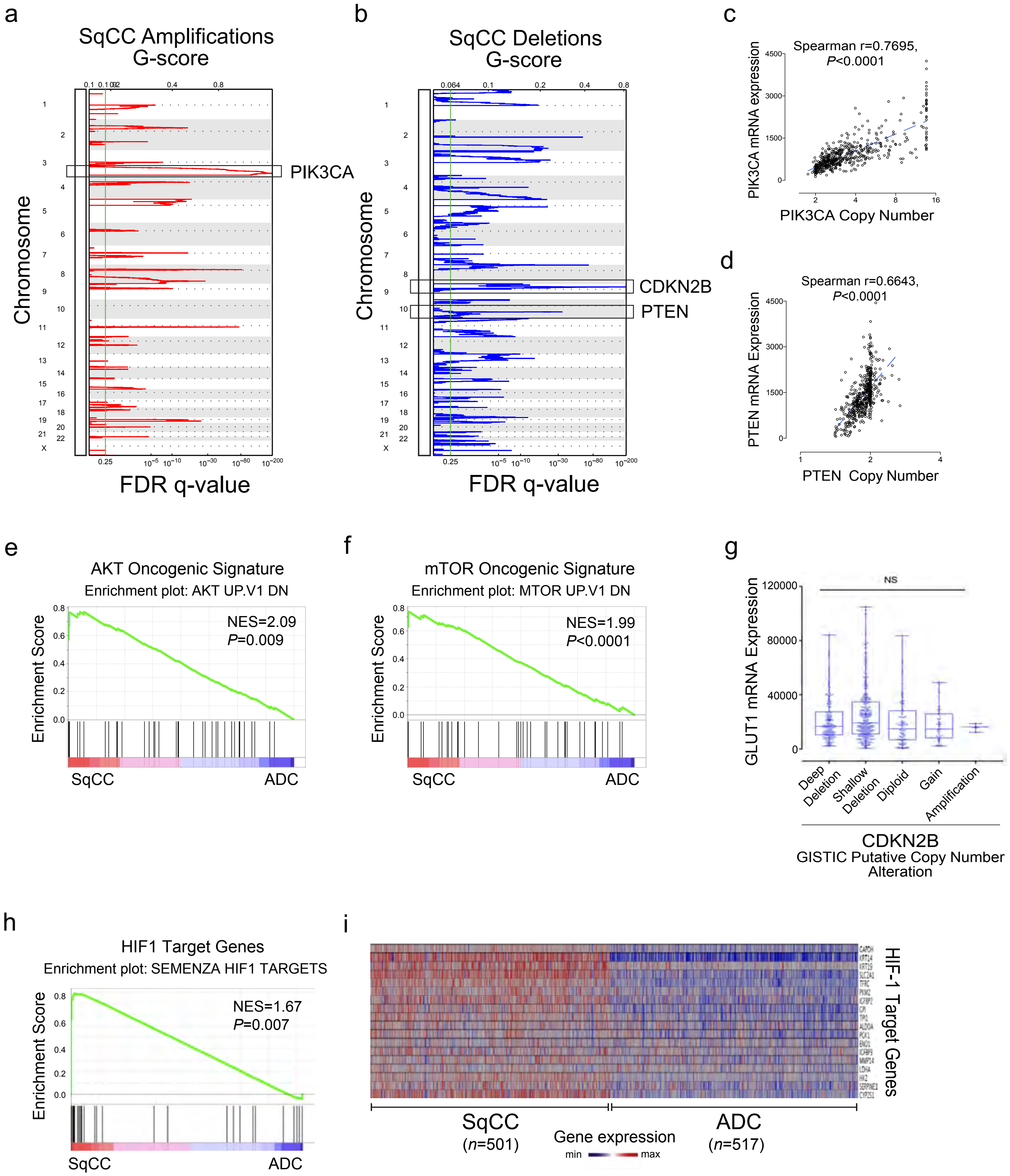

a
SqCC Amplifications
G-score
PIK3CA
Chromosome
FDR q-value
b
SqCC Deletions
G-score
CDKN2B
PTEN
Chromosome
FDR q-value
c
Spearman r=0.7695,
P<0.0001
PIK3CA mRNA expression
PIK3CA Copy Number
d
Spearman r=0.6643,
P<0.0001
PTEN mRNA Expression
PTEN Copy Number
e
AKT Oncogenic Signature
Enrichment plot: AKT UP.V1 DN
NES=2.09
P=0.009
Enrichment Score
SqCC
ADC
f
mTOR Oncogenic Signature
Enrichment plot: MTOR UP.V1 DN
NES=1.99
P<0.0001
Enrichment Score
SqCC
ADC
g
NS
GLUT1 mRNA Expression
Deep Deletion
Shallow Deletion
Diploid
Gain
Amplification
CDKN2B
GISTIC Putative Copy Number Alteration
h
HIF1 Target Genes
Enrichment plot: SEMENZA HIF1 TARGETS
NES=1.67
P=0.007
Enrichment Score
SqCC
ADC
i
HIF-1 Target Genes
SqCC
(n=501)
Gene expression
min
max
ADC
(n=517)

Supplementary Figure 14 Elevated PIK3/AKT/HIF-1 pathways in TCGA patient samples. (**a**, **b**) GISTIC analysis of TCGA SqCC tumor samples shows the landscape of genomic amplifications (**a**) and deletions (**b**). (**c**, **d**) Correlation analysis of mRNA expression (normalized TPM) and copy number variation of PIK3CA (**c**) and PTEN (**d**) in the TCGA cohort of lung SqCC (n=498). Spearman r is provided for correlation.(**e**, **f**) GSEA mountain plots for the enrichment of AKT and mTOR oncogenic signatures in SqCC compared to ADC. NES, normalized enrichment score. (**g**) GLUT1 mRNA expression (normalized TPM) and the genomic loss of CDKN2B (p15) in TCGA SqCC patients. Each dot represents one SqCC patient (n=501). n.s., not significant. (**h**) GSEA mountain plot for the enrichment of HIF-1 target gene expression in SqCC compared to ADC. NES, normalized enrichment score. (**i**) Heatmap depicting analysis of HIF-1 target gene expression profiles of the TCGA SqCC and ADC patients.

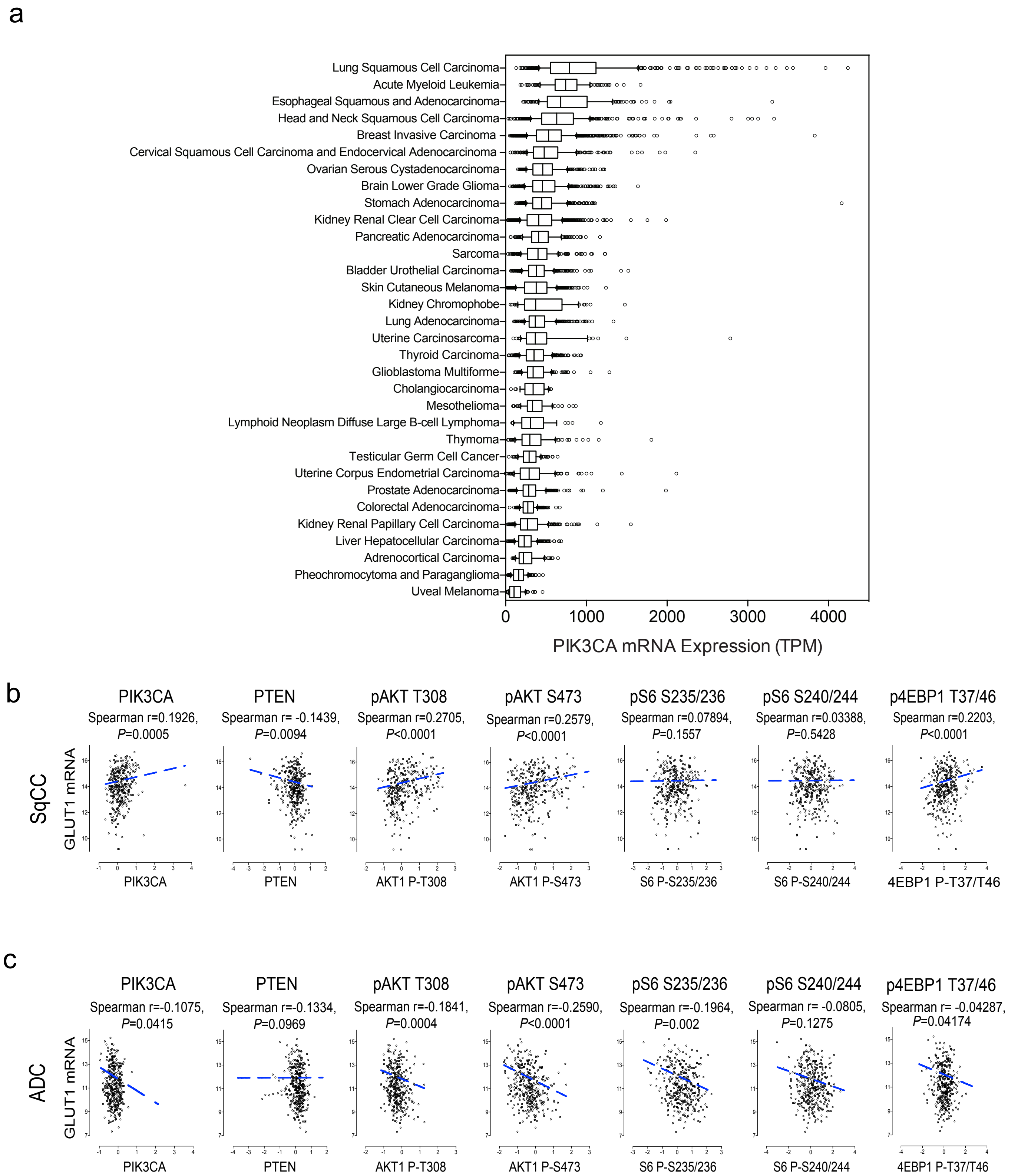

Supplementary Figure 15 Elevated PI3K/AKT signaling is associated with GLUT1 expression in TCGA lung SqCC tumors. (**a**) Pan-TCGA analysis of PIK3CA mRNA expression. Boxes represent the median the interquartile range, error bars are drawn from the 10th to the 90th percentile, outliers are denoted with open circles. (**b**, **c**) Correlative analysis of GLUT1 mRNA expression (nprmalized TPMA) and RPPA PI3K pathway signaling protein expression in the TCGA cohorts of lung SqCC, n=328 (**b**) and ADC, n=365 (**c**). Spearman correlation coefficients are presented with probabilities for correlations.

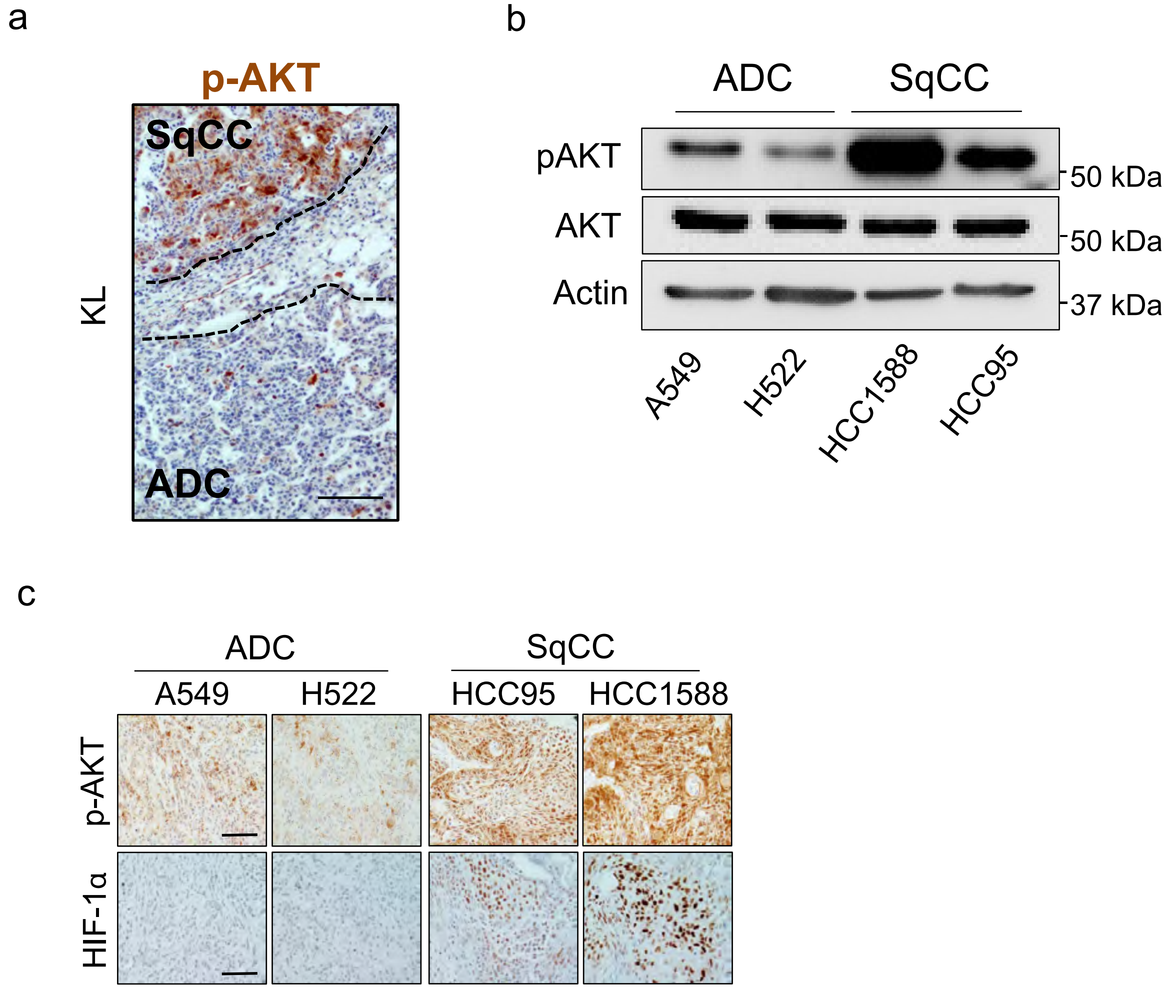

Supplementary Figure 16 Elevated PIK3/AKT/HIF-1 pathways in lung SqCC cell lines and xenograft tumors. (**a**) IHC analysis of p-AKT in KL mouse model. Scale bar, 100 μm. (**b**) Immunoblot analysis of p-AKT level in ADC cell lines, A549 and H522, and SqCC cell lines, HCC1588 and HCC95. (**c**) IHC analysis of p-AKT and HIF-1 expression in ADC (A549 and H522) and SqCC (HCC95 and HCC1588) cell line-derived xenograft tumors. Scale bar, 100 μm.

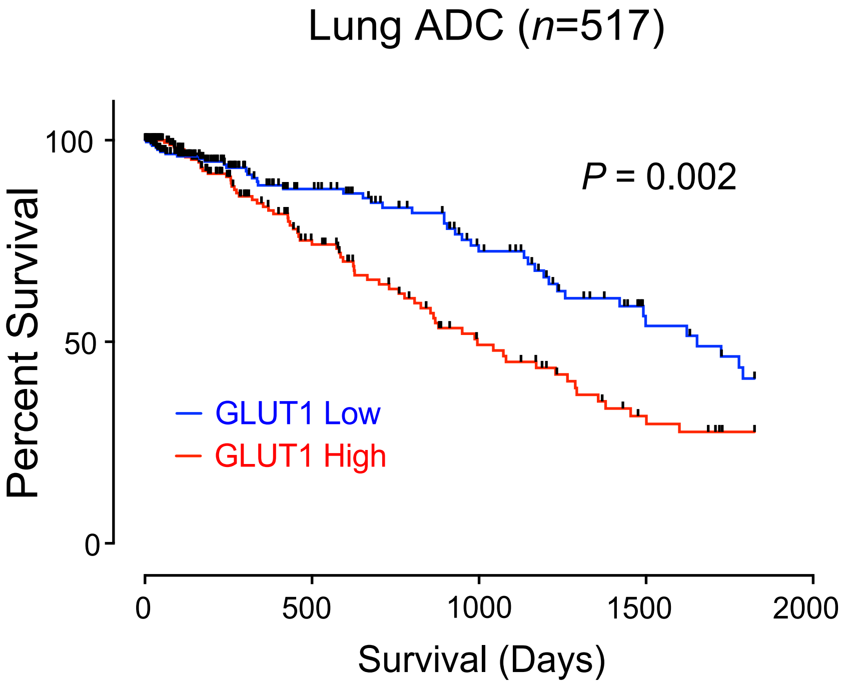

Supplementary Figure 17 High GLUT1 expression is associated poor survival in TCGA lung ADC tumors. Kaplan-Meier 5-year survival analysis comparing GLUT1 high and low expressing patients in the TCGA lung ADC cohort. GLUT1 high and low groups were separated by the median expression. Significance was determined with log rank test. P=0.002; HR, 1.74.

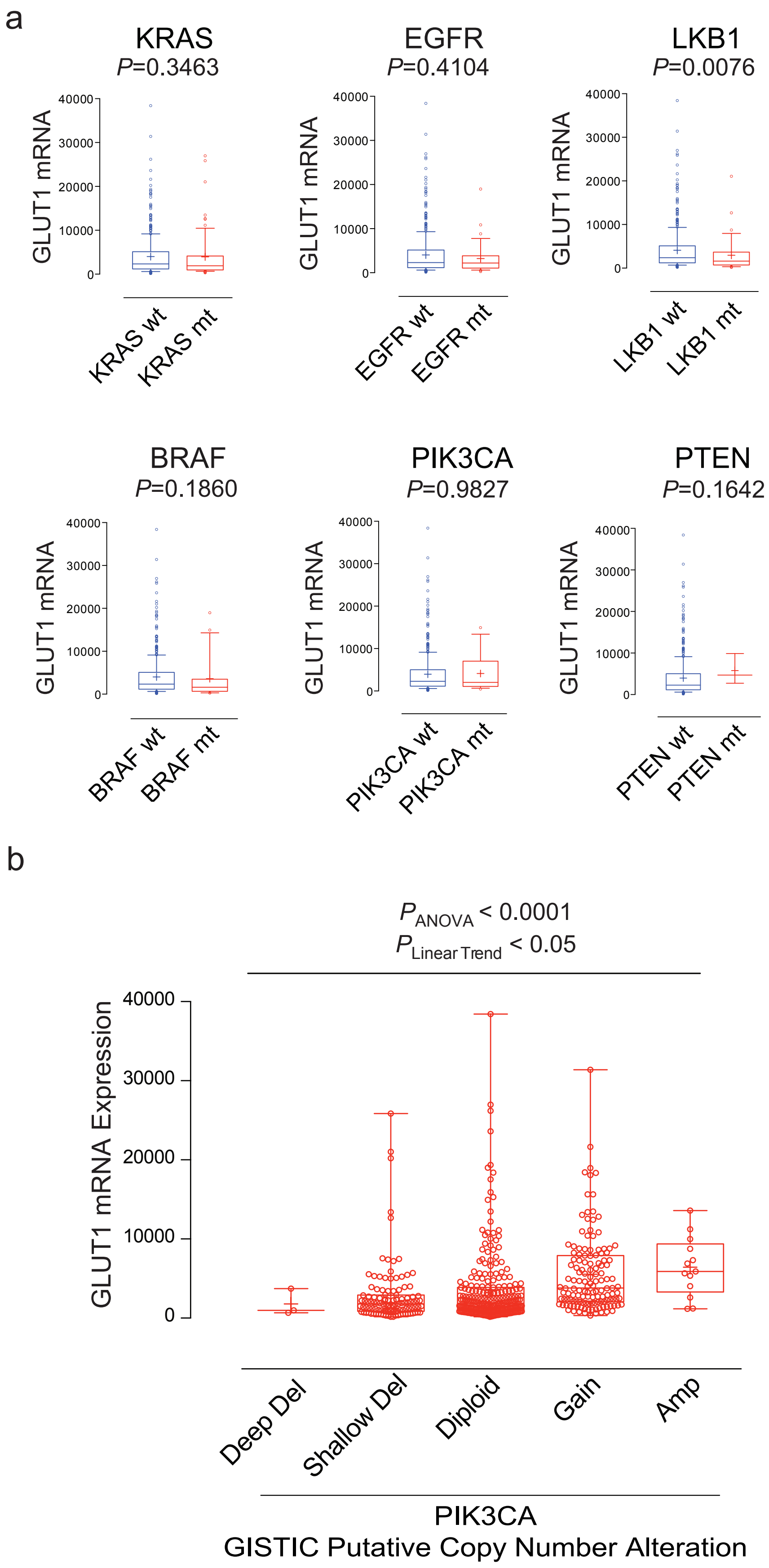
a
KRAS
P=0.3463
EGFR
P=0.4104
LKB1
P=0.0076
GLUT1 mRNA
KRAS wt
KRAS mt
EGFR wt
EGFR mt
LKB1 wt
LKB1 mt
BRAF
P=0.1860
PIK3CA
P=0.9827
PTEN
P=0.1642
BRAF wt
BRAF mt
PIK3CA wt
PIK3CA mt
PTEN wt
PTEN mt
b
P ANOVA < 0.0001
P Linear Trend < 0.05
GLUT1 mRNA Expression
Deep Del
Shallow Del
Diploid
Gain
Amp
PIK3CA
GISTIC Putative Copy Number Alteration

Supplementary Figure 18 GLUT1 mRNA expression in Kras or EGFR mutant lung ADC tumors. (**a**) TCGA mRNA sequencing analysis of GLUT1 mRNA expression (normalized TPM) in ADC patients (n=518) with KRAS activating mutations (n=73), EGFR mutations (n=32), LKB1 mutations (n=40), BRAF mutations (n=22), PIK3CA mutations (n=15), and PTEN mutations (n=3). Boxes represent the median the interquartile range, error bars are drawn from the 10th to the 90th percentile, outliers are denoted with open circles. Mann Whitney u-test, ns, not significant. (**b**) Analysis of TCGA GLUT1 mRNA expression (normalized TPM) and PIK3CA genomic copy number alteration profiles. Each dot represents one ADC patient (n=518). Boxes represent the interquartile range and whiskers are drawn to the minimum and maximum. Kruskal-Wallis non-parametric ANOVA.

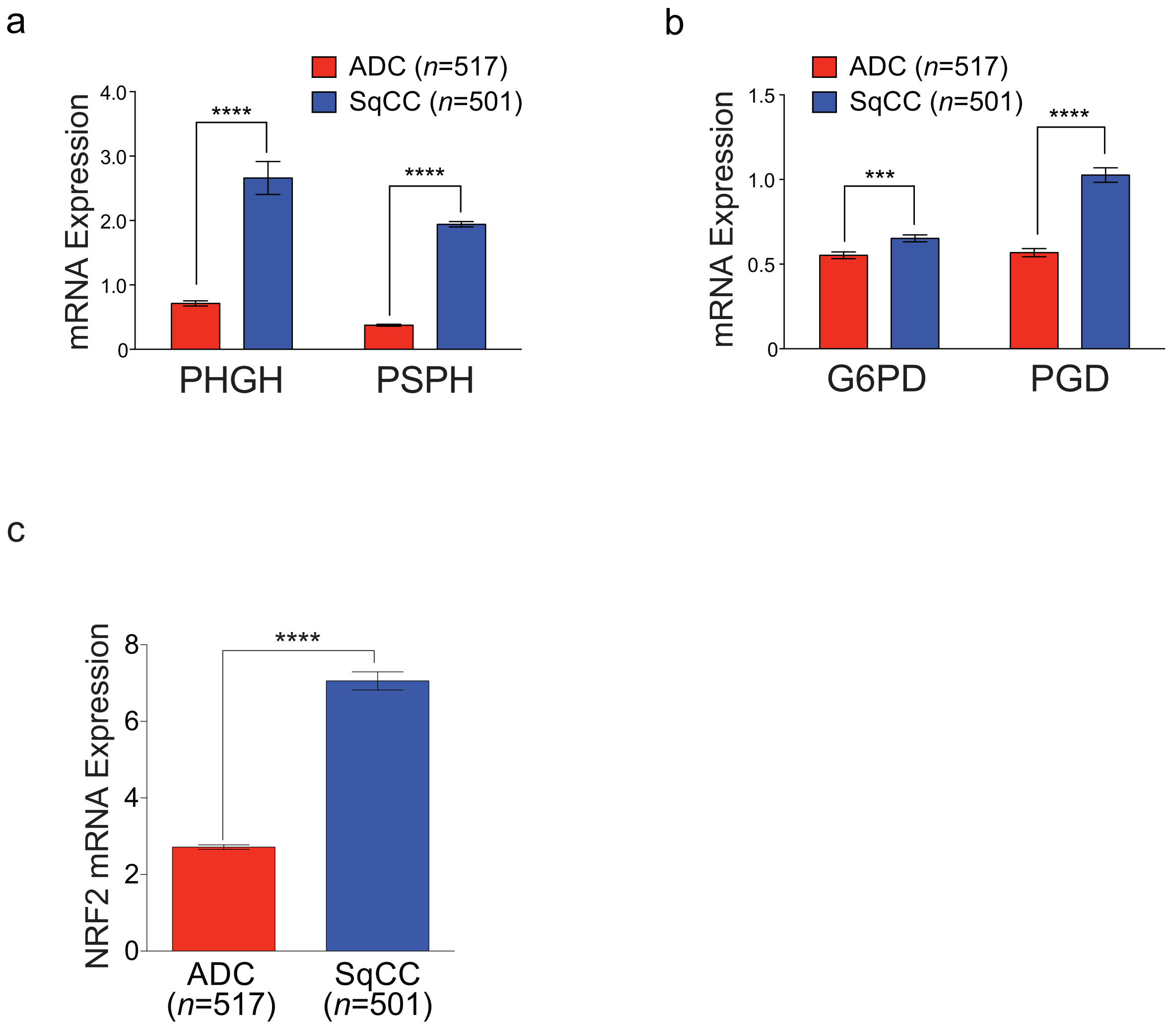

Supplementary Figure 19 Serine biosynthesis and pentose phosphate pathway in lung SqCC. (**a**, **b**) TCGA mRNA sequencing expression (normalized TPM) of serine biosynthesis genes (**a**) and pentose phosphate pathway genes (**b**) in TCGA ADC and SqCC tumor samples. Error bars represent the mean s.e.m. Mann Whitney u-test. ****P<0.0001, ***P<0.001. (c) Nrf2 mRNA expression in TCGA ADC and SqCC tumor samples. Mann Whitney u-test. ****P<0.0001.

Lung ADC (*n*=517)

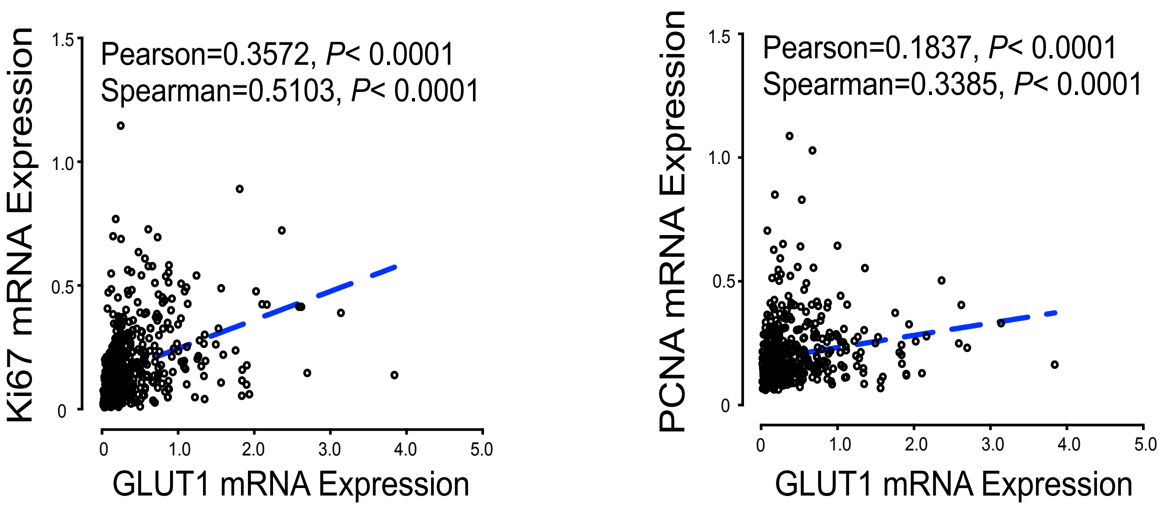

Supplementary Figure 20 High GLUT1 expression is correlated with proliferation markers in lung ADC. Correlative analysis of GLUT1 mRNA expression with proliferative markers, Ki67 (left) and PCNA (right), mRNA expressions in the TCGA cohort of ADC (n=517). Pearson and Spearman R-values and probabilities are presented for correlations. Gene expression values are normalized TPM.

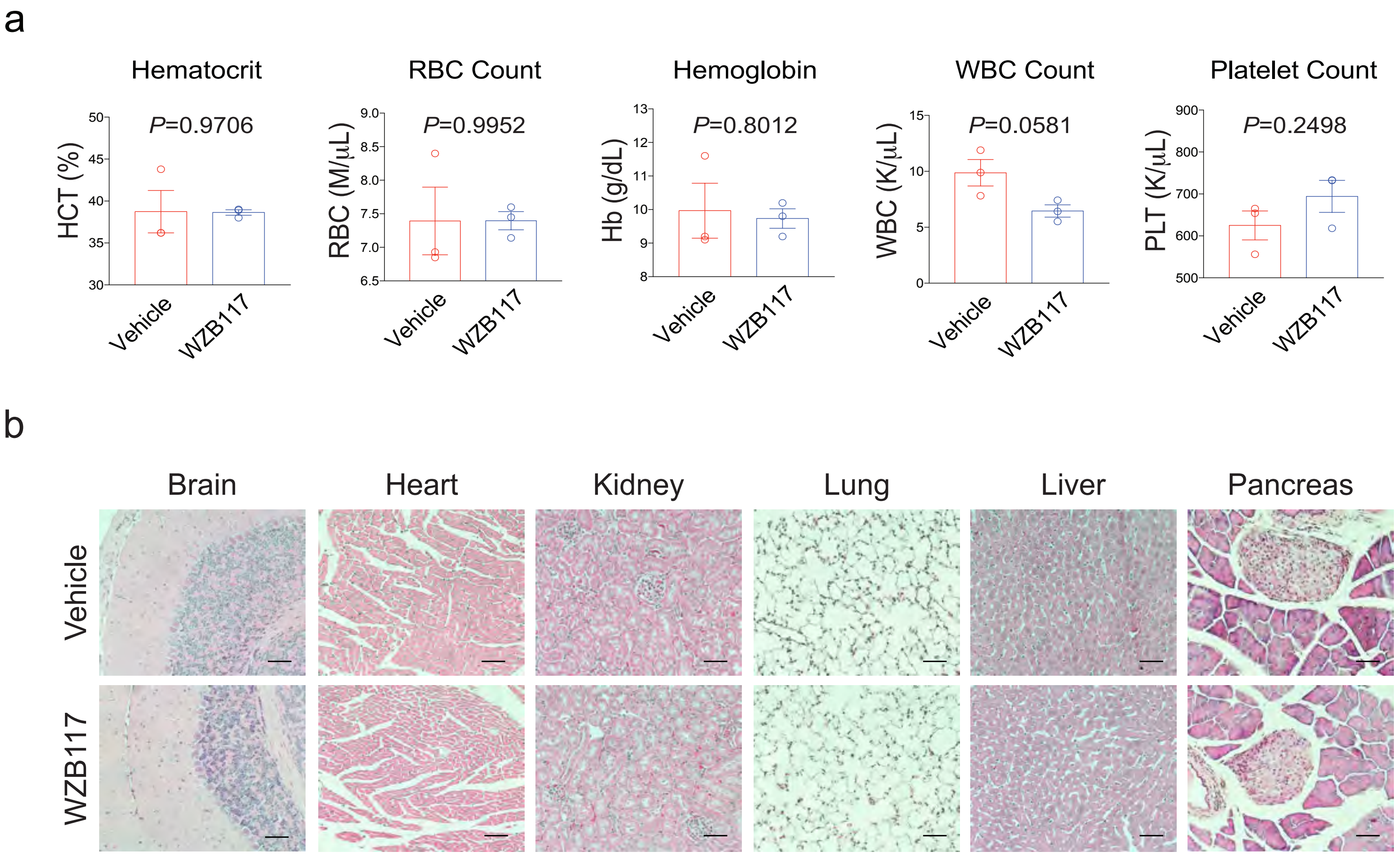

Supplementary Figure 21 Hematological and histological profiles of WZB117-treated mice. (**a**) Comparison of hematocrit, RBC count, WBC count, platelet content, and hemoglobin in vehicle (n=3) and WZB117 (n=3) treated mice after 3 weeks of treatment (10mg/kg daily). Error bars represent mean ± s.e.m. Two-tailed t-test. (**b**) Comparison of representative H&E stained tissues of brain, muscle, lung, heart, liver, kidney, and pancreas of vehicle (n=3) and WZB117 (n=3) treated mice after 3 weeks of treatment (10mg/kg daily). Scale bars, 100μM.

Related to Fig. 2b

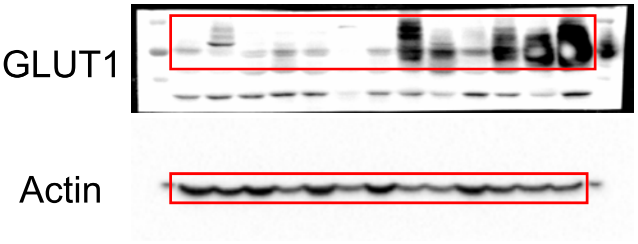

Related to Fig. 3a

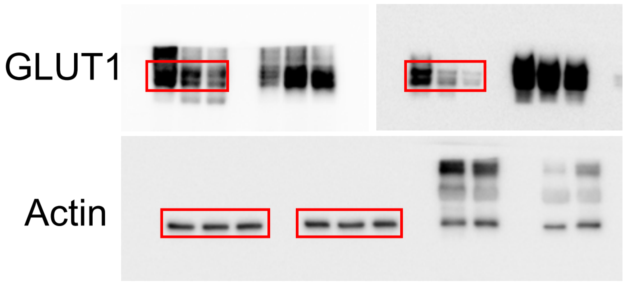

Related to Fig. 7d

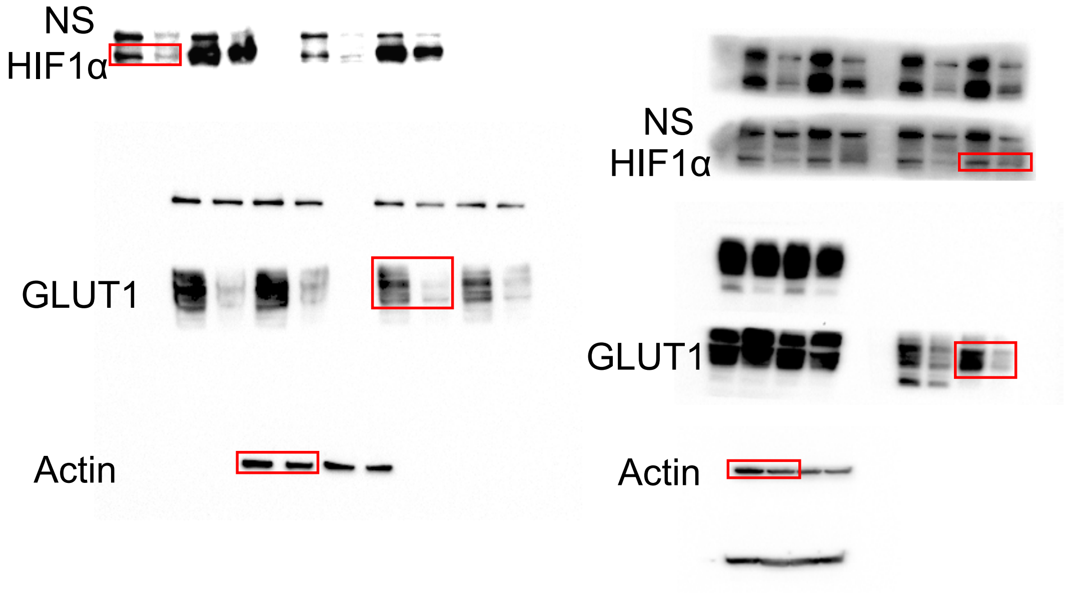

Related to Supplementary Fig. 6a

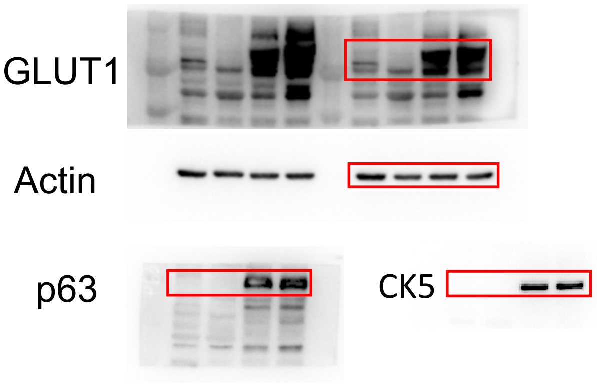

Related to Supplementary Fig. 7a

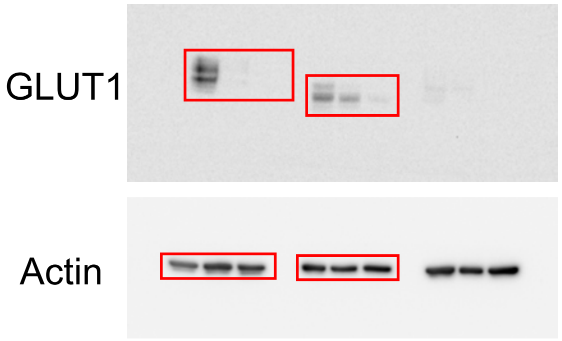

Related to Supplementary Fig. 8a

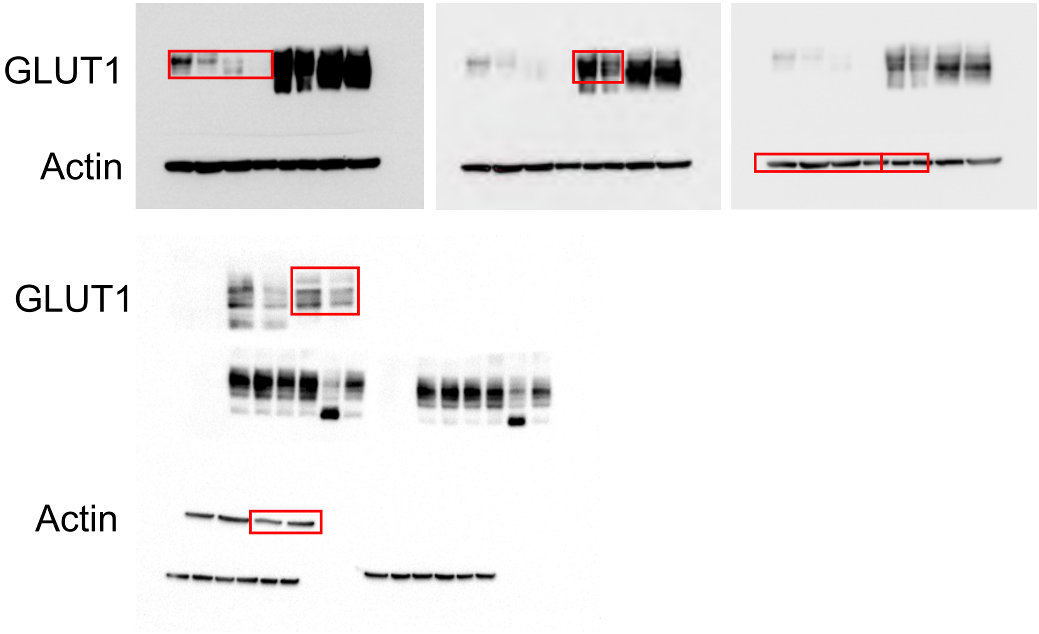

Related to Supplementary Fig. 8d

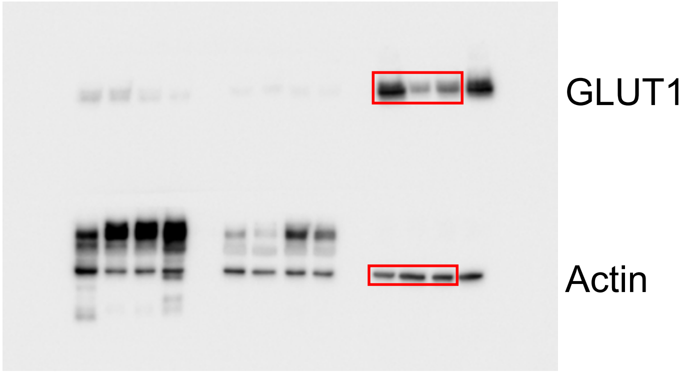

Supplementary Figure 22 Unprocessed immunoblots Unprocessed, uncropped images of immunoblots are presented with reference to the original figures for each blot.

Supplementary Table 1: Clinical characteristics of NSCLC patient cohort

ID	Histology	Sex	Age	Smoking status	PY	Pathologic stage	Tumor cell differentiation	SUVmax	GLUT1 IHC Intensity score	GLUT1 IHC positive area (%)	mRNA tumor	mRNA normal
1	ADC (A100)	M	65	current	30	Ia	well	4.8	0	0	23.75	1.07
2	ADC (A80 P10 L10)	M	64	current	45	Ib	well	6	0	0	6.59	3.39
3	ADC (P100)	M	80	former	33	Ia	moderately	3.2	0	0	5.1	1.97
4	ADC (L90 A10 Muc)	M	55	former	30	Ib	moderately	1.9	0	0	10.48	4.92
5	ADC (A70 P30)	F	68	never	0	Ib	moderately	4	0	0	5.24	2.27
6	ADC (A60 P40)	F	74	never	0	V	moderately	5.6	0	0	6.06	13.55
7	ADC (A80 P19 MP1)	F	76	never	0	Ib	moderately	7.5	2	1	15.67	10.63
8	ADC (P80 A10 L10)	F	71	never	0	Ib	well	10.9	1	5	5.06	1.82
9	ADC (P85 MP15)	F	58	never	0	IIIa	moderately	5.4	2	10	3.68	1
10	ADC (A50 MP30 P20)	M	61	former	20	IIa	well	11.7	2	20	13.27	2.38
11	SqCC	F	56	former	10	Ia	moderately	11	3	20	117.78	24.08
12	SqCC	M	77	current	50	Ia	moderately	7.5	3	30	17.75	3.23
13	SqCC	M	72	current	50	IIa	moderately	11.9	3	30	238.86	3.23
14	SqCC	M	57	former	35	IIa	moderately	18.9	3	40	17.88	1.68
15	ADC (A90 S10)	M	63	current	40	IIa	moderately	12.8	3	40	85.04	1.95
16	ADC (A50 MP40 P10)	M	55	current	40	IIIa	moderately	16.3	3	40	28.25	57.28
17	SqCC	M	61	former	35	IIa	moderately	8.9	3	45	91.77	3.92
18	ADC (A70 P30)	F	69	never	0	Ia	moderately	9.2	3	50	8	66.72
19	SqCC	M	74	former	40	Ib	poorly	38.6	3	50	11.55	7.21
20	ADC (A95 S5 feta)	M	54	current	30	Ib	moderately	13.1	1	60	12.04	15.03
21	SqCC	F	70	current	20	Ia	moderately	9.3	3	60	235.57	4.23
22	SqCC	M	77	former	25	Ib	moderately	19.1	3	60	151.17	33.13
23	SqCC	F	69	never	0	IIa	moderately	37.1	3	60	5.86	
24	SqCC	M	59	former	40	IIa	moderately	12.5	3	60	10.85	3.97
25	SqCC	M	63	current	75	IIIa	moderately	12.1	3	60	276.28	1.58
26	SqCC	M	70	former	75	IIb	poorly	17.7	3	65	120.26	1.73
27	SqCC	M	71	current	50	IIb	moderately	14.7	3	80	380.04	2.27
28	ADC (A95 S5 muc)	M	65	former	30	IIb	moderately		3	80	65.8	1.65
29	SqCC	M	61	current	35	IIb	moderately	16.4	3	80	152.22	1.47
30	SqCC	M	64	former	30	Ia	moderately	8.9	3	90	205.07	2.31
31	SqCC	M	66	former	38	IIa	moderately	21.5	2	100	140.07	1.69
32	SqCC	M	64	current	20	Ia	moderately	6.7	3	100	60.97	3.73
33	SqCC	M	57	current	60	Ia	moderately	7.4	3	100	167.73	
34	SqCC	M	71	former	6	Ib	moderately	15.2	3	100	44.32	38.32
35	SqCC	M	50	former	50	Ib	moderately	10.3	3	100	178.53	1.99
36	SqCC	M	63	former	60	IIa	poorly	20.3	3	100	58.08	1.16

Abbreviations: PY, pack-year of smoking; IHC, immunohistochemistry; ADC, adenocarcinoma; SqCC, squamous cell carcinoma.
A, Acinar ADC; P, Papillary ADC; L, Lepidic ADC; MP, Micropapillary ADC; S, Solid ADC; Muc, Mucinous; feta, fetal ADC
SUVmax by 18F-FDG PET scan.
GLUT1 mRNA is relative expression.

Supplementary Table 2: Clinical characteristics of patient cohort used to establish PDX model

PDX No.	Histology	Age	Sex	Stage	Clinical Outcome	Source	Disease Free (M)	KRAS*	EGFR*
E784	SqCC	60s	M	IA	no recurrence	tumor	36.2		
E1110	SqCC	70s	M	IIA	recurrence	tumor	13.5		
E1034	SqCC	50s	M	IB	no recurrence	tumor	11.9		
E1036	SqCC	80s	M	IIIB	recurrence	tumor	5.6		
E1088	SqCC	50s	M	IIIA	no recurrence	tumor	5.7		
E1024	SqCC	50s	M	IB	no recurrence	tumor	6.2		
E1069	SqCC	60s	M	IIB	no recurrence	tumor	6.8		
E1124	ADC	50s	M	IV	metastasis	tumor	0.0		
E851	ADC	60s	F	IV	metastasis	pl. effusion	0.0	G12V	wt
E1032	ADC	60s	M	IV	metastasis	pl. effusion	0.0	wt	wt
E994	ADC	30s	M	IV	metastasis	pl. effusion	0.0	wt	ex19del
E815	ADC	60s	M	IIB	recurrence	tumor	6.9	wt	wt
E1104	ADC	70s	M	IV	metastasis	tumor	0.0	wt	wt
E1092	ADC	70s	M	IB	no recurrence	tumor	15.0	wt	ex19del

* KRAS and EGFR mutational statuses are not available from all SqCC patients and ADC patient E1124

Supplementary Table 3: Mutational status of NSCLC cell lines

Group	Cell line	P53	KRAS	EGFR	LKB1	PIK3CA	PTEN
ADC	**H23**	M247I	G12C	WT	W332STOP	WT	WT
	**A549**	WT	G12S	WT	Del, Q37STOP	WT	WT
	**A427**	WT	G12D	WT	WT	WT	WT
	**H1299**	Del	WT	WT	WT	Del	WT
	**H522**	Del	WT	WT	WT	Amp	WT
	**H1568**	H179R	WT	WT	Del	WT	WT
	**H1155**	R273H	61H`	WT	WT	D843E	R233STOP
SqCC	**HCC1588**	WT	G12D	WT	WT	WT	WT
	**H1897**	NA	NA	NA	NA	NA	NA
	**HCC2450**	NA	NA	NA	NA	NA	NA
	**H1869**	M247I	WT	WT	WT	WT	WT
	**HCC95**	WT	WT	WT	Del	Amp	WT
	**HCC15**	D259V	WT	WT	Del	Del	WT

Supplementary Table 4: qRT-PCR primer sequences for hGLUT1-14 (hSLC2A1-14)

Gene	Orientation	Sequences (5'-3')
hSLC2A1	Forward	ACCTCAAATTTCATTGTGGG
	Reverse	GAAGATGAAGAACAGAACCAG
hSLC2A2	Forward	AGAAGATTAGACTTGGACTCTC
	Reverse	GTGACCTTATCTTCTGTCATTG
hSLC2A3	Forward	GGATAACTATAATGGGATGAGC
	Reverse	CCACAATAAACCAGGGAATG
hSLC2A4	Forward	TCCTTCCTCATTGGTATCATC
	Reverse	CCAAGGATGAGCATTTCATAG
hSLC2A5	Forward	TAAATTTGGCAGAAAAGGGG
	Reverse	ATATTCCCACCAAAAGTCTG
hSLC2A6	Forward	TCTTCATCATGGGCTACG
	Reverse	CGCGAAGAAGAAGAAAGG
hSLC2A7	Forward	AATGCGATCAACTACTATGC
	Reverse	CGTTACATATTGGGAGTGAG
hSLC2A8	Forward	CAAGTTCAAGGACAGCAG
	Reverse	TCTGTCCATGATGAGAGC
hSLC2A9	Forward	AAAAGTGAACCATGAAGCTC
	Reverse	GTCCAATTTCTTTTTCGCTG
hSLC2A10	Forward	GATCTCATTGGCACCATC
	Reverse	AACTGCTGGTCTATCTCTG
hSLC2A11	Forward	GGATTTCCCTTTATCATGGAG
	Reverse	AGATCTCTTGGAAGGTCTTG
hSLC2A12	Forward	GACTGTAACTGATCTTATTGGC
	Reverse	ATTTGTTCCAAAGAGCATCC
hSLC2A13	Forward	ATGGAGCTTTCTTCCTCTATG
	Reverse	CACATGTACATAGCCTGTTG
hSLC2A14	Forward	CTGTGTTCTATTACTCAACAGG
	Reverse	GATAGTATTAACCACACCCG
hACTIN	Forward	GTGACAGCAGTCGGTTGGAG
	Reverse	AGGACTGGGCCATTCTCCTT

Supplementary Table 5: shRNA and siRNA Targeting Sequences

Gene	RNA	Sequence
HIF-1A	shHIF-1α	CCGGTGCTCTTTGTGGTTGGATCTACTCGAGTAGATCCAACCACAAAGAGCATTTTT
SLC2A1	shGLUT1 #1	CCGGGCCACACTATTACCATGAGAACTCGAGTTCTCATGGTAATAGTGTGGCTTTTTG
SLC2A1	shGLUT1 #2	CCGGTGCTCTTTGTGGTTGGATCTACTCGAGTAGATCCAACCACAAAGAGCATTTTT
SLC2A1	siRNA	GUAUGUGGGUGAAGUGUCA
SLC2A1	siRNA	AGACAUGGGUCCACCGCUA
SLC2A1	siRNA	CAAAUUUCAUUGUGGGCAU
SLC2A1	siRNA	ACUCAUGACCAUCGCGCUA
